# Clinical significance of gut microbiota-derived metabolite trimethylamine N-oxide in patients with systemic lupus erythematosus

**DOI:** 10.1038/s41598-026-53011-7

**Published:** 2026-05-22

**Authors:** Jiwon Yang, Youngjae Park, Se Gwang Jang, Min-Jung Park, Su-Jin Moon, Hyung-Kyoon Choi, Seung-Ki Kwok

**Affiliations:** 1 Division of Rheumatology, Department of Internal Medicine, Seoul St. Mary’s Hospital, College of Medicine, The Catholic University of Korea, Seoul, Republic of Korea; 2 The Rheumatism Research Center, Catholic Research Institute of Medical Science, The Catholic University of Korea, Seoul, Republic of Korea; 3 Division of Rheumatology, Department of Internal Medicine, Yeouido St. Mary’s Hospital, College of Medicine, The Catholic University of Korea, Seoul, Republic of Korea; 4 College of Pharmacy, Chung-Ang University, Seoul, Republic of Korea

**Keywords:** Cardiovascular disease, Gut microbiome, Metabolite, Organ damage, Systemic lupus erythematosus, Trimethylamine N-oxide, Biomarkers, Diseases, Immunology, Medical research, Rheumatology

## Abstract

Trimethylamine N-oxide (TMAO), a gut microbiota-derived metabolite, is associated with cardiovascular disease (CVD) via pro-inflammatory and pro-atherogenic mechanisms. Systemic lupus erythematosus (SLE) is a systemic autoimmune disease with a significantly increased risk of CVD, however, the role of TMAO in SLE remains unclear. This study aimed to assess the clinical significance of TMAO in patients with SLE, including analyses of precursor metabolites and gut microbiome. A total of 207 participants were enrolled, including 157 patients with SLE and 50 healthy controls. Serum TMAO levels were measured using ELISA, fecal precursor metabolites including trimethylamine (TMA) were quantified by ^1^H-NMR spectroscopy, and gut microbiota composition was assessed using 16S rRNA sequencing. SLE patients with CVD had significantly higher TMAO levels than HC (*P* = 0.028) and SLE patients without CVD (*P* = 0.004). Serum TMAO (*P* = 0.025) and fecal TMA (*P* = 0.032) levels were positively correlated with the Systemic Lupus International Collaborating Clinics/American College of Rheumatology (SLICC/ACR) damage index. Serum TMAO was negatively correlated with steroid dose (*P* = 0.038). Distinct gut microbiota compositions were observed between SLE patients with and without CVD. TMAO is associated with cardiovascular disease and cumulative organ damage in SLE, potentially mediated by gut microbiome alterations and modulated by steroid therapy.

**Supplementary Information:**

The online version contains supplementary material available at 10.1038/s41598-026-53011-7.

## Introduction

Systemic lupus erythematosus (SLE) is characterized by systemic inflammation and a markedly elevated risk of cardiovascular disease (CVD), including accelerated atherosclerosis and premature vascular events, compared with the general population^1,2^. This heightened cardiovascular risk significantly contributes to patient morbidity and mortality and is only partially explained by traditional risk factors, posing a major clinical challenge^3,4^. As traditional factors fall short of fully explaining this risk, emerging evidence suggests that gut microbiota-derived metabolites may play a pivotal role in modulating these cardiovascular and inflammatory pathways^5–7^.

Among these metabolites, Trimethylamine N-oxide (TMAO) has garnered significant attention as a biologically plausible mediator. TMAO is the oxidized product of trimethylamine (TMA), a metabolite produced by the gut microbiota from dietary choline, carnitine, and betaine^5,7^. These are metabolized by microbial TMA lyases (*cutC*/*D* system) to form TMA in the gut^5,8,9^. Once formed, TMA is absorbed into the circulation and transported to the liver, where it is oxidized to TMAO by flavin-containing monooxygenases (FMO), primarily FMO3^5,7,10^. Based on current evidence, this gut–liver axis is considered the primary and most well-established endogenous pathway for TMAO production^5,7^. In addition to oxidation in the liver, fecal TMA can be oxidized to fecal TMAO in the gut by TMAO monooxygenase. Fecal TMAO may be reduced back to TMA by TMAO reductase or further metabolized to dimethylamine (DMA) by TMAO demethylase. Circulating TMAO has been extensively reported to be closely associated with CVD^5,7^. Recent studies have suggested that TMAO contributes to cardiovascular complications by promoting inflammatory signaling, endothelial dysfunction, and platelet hyperreactivity^5,11^. Elevated serum TMAO levels have been independently linked to an increased risk of myocardial infarction, stroke, and heart failure^7,12^.

Given the established pro-inflammatory nature of TMAO and the frequent gut dysbiosis observed in SLE patients^13–15^, it is highly plausible that TMAO contributes to the accelerated cardiovascular complications in this population^16^. However, studies investigating the role of TMAO in SLE remain limited. González-Correa et al. demonstrated that elevated TMAO levels exacerbate autoimmunity and vascular dysfunction in lupus-prone mice, whereas inhibition of TMA formation using 3,3-dimethyl-1-butanol significantly attenuated disease severity and endothelial injury in vivo^17^. Li et al. reported that the serum TMAO levels were significantly higher in patients with SLE compared to those in healthy controls (HCs) based on untargeted metabolomic analysis^18^. Although the pro-inflammatory properties of TMAO have been clinically investigated in other autoimmune diseases, including rheumatoid arthritis^19^, systemic sclerosis^20,21^, and psoriatic arthritis^22^, evidence regarding its role in SLE remains scarce. Particularly, no studies to date have comprehensively examined the association between TMAO and cardiovascular complications in patients with SLE.

Thus, we conducted an observational cross-sectional cohort study to assess the clinical significance of TMAO in patients with SLE. Specifically, we examined the association between TMAO levels and cardiovascular complications. Furthermore, we investigated its relationships with other clinical parameters, such as disease activity, organ damage index, and medication use. Additionally, analyses of fecal precursor metabolites and gut microbiota composition were conducted to better elucidate the potential mechanistic link between gut microbiota-derived metabolites and systemic inflammation in SLE.

## Methods

### Study participants and sample collection

A total of 207 participants, comprising 157 patients with SLE and 50 HCs, were recruited from Seoul St. Mary’s Hospital and Yeouido St. Mary’s Hospital, university-affiliated tertiary and secondary referral centers in Korea. patients with SLE were eligible for inclusion if they were between 19 and 65 years of age and met the 2019 European League Against Rheumatism/ACR classification criteria for SLE^23^. HCs were individuals within the same age range who had no history of SLE. Exclusion criteria for both groups included the presence of other autoimmune diseases, inability to provide a fecal sample due to medical, surgical, or psychiatric conditions, current use of antibiotics or probiotics, and pregnancy or lactation. HCs were additionally excluded if they had any chronic diseases requiring medication that could influence gut microbiota composition or systemic inflammation.

Serum and fecal samples were collected simultaneously at a single time point from each participant after ≥ 8 h of fasting. Serum was isolated from peripheral blood samples by centrifugation for analysis. Serum and fecal samples were refrigerated immediately after collection and transported to the laboratory on the same day. Upon arrival, all samples were stored at − 70 °C until analysis.

Data on patient demographics, clinical characteristics, and disease-specific metrics were collected at the time of serum and fecal sample collection. These included age, sex, BMI, SLE disease duration, complement 3 and 4 (C3 and C4) levels, anti-double stranded DNA (anti-DNA) antibody levels, the SLEDAI-2 K score^24^, the SLICC/ACR DI^25^, presence of lupus nephritis, presence of comorbid CVD, concurrent use of steroids and hydroxychloroquine, and steroid dose. CVD was defined as the presence of clinically diagnosed coronary artery disease, stroke, heart failure, hypertension, or dyslipidemia. All steroid doses are expressed as prednisolone equivalents. This study was approved by the Institutional Review Board of Seoul St. Mary’s Hospital and Yeouido St. Mary’s Hospital, Catholic University of Korea (XC23TNDI0055). All patients provided written informed consent before participation in accordance with the principles of the Declaration of Helsinki. All methods were performed in accordance with the relevant guidelines and regulations.

### Enzyme-linked immunosorbent assay (ELISA)

The serum TMAO levels were measured using ELISA kits, and the procedures were performed according to the manufacturer’s instructions (catalog number: MBS7269386; MyBioSource, San Diego, CA, USA).

### Nuclear magnetic resonance spectroscopy

The fecal levels of choline, TMA, TMAO, and DMA were analyzed in individual fecal samples using proton nuclear magnetic resonance (^1^H-NMR) spectroscopy. Spectra were acquired at 600.13 MHz on a Bruker Avance III 600 spectrometer (Bruker, Karlsruhe, Germany). One-dimensional ^1^H pulse experiments were performed at 298 K using a nuclear Overhauser effect spectroscopy pre-saturation sequence to suppress the residual water signal. Spectral binning and normalization of the ^1^H-NMR data were conducted using Chenomx NMR Suite software (version 8.2; Chenomx Inc., Edmonton, AB, Canada).

### Microbial DNA extraction and 16S rRNA sequencing

Microbial DNA was extracted from fecal samples using the SPINeasy DNA Pro Kit (MP Biomedicals, Irvine, CA, USA) or the Maxwell RSC PureFood Kit (Promega, Madison, WI, USA) following the manufacturers’ protocols. The V3–V4 regions of the 16S rRNA gene were amplified using primers 341 F and 805R, and polymerase chain reaction products were purified, quantified with the QuantiFluor™ dsDNA system (Promega), and pooled. Sequencing was performed on the Illumina MiSeq platform (2 × 300 bp paired-end reads) at CJ Bioscience, Inc. (Seoul, Korea). Additionally, microbial functional profiles were predicted using the PICRUSt2 pipeline (v2.4.1)^26^ through QIIME2 (v2021.11) and CJ Bioscience’s EzBioCloud 16S-based MTP analysis platform. To assess the reliability of the metagenomic predictions, the Nearest Sequenced Taxon Index (NSTI) was calculated for each sample. The mean NSTI across all samples was 0.021 ± 0.013 (median 0.017; IQR 0.010; range 0.005–0.094), suggesting that most amplicon sequence variants were closely matched to sequenced reference genomes and that the inferred functional profiles were reasonably reliable. Functional annotations were assigned based on the KEGG Orthology (KO) and Enzyme Commission (EC) databases^27^.

### Statistical analysis

The distribution of continuous variables was assessed using the Kolmogorov–Smirnov test. Variables with a normal distribution were expressed as means ± standard errors of the mean (SEMs), whereas non-normally distributed variables were presented as median and IQR. Categorical variables were reported as frequencies and percentages. Comparisons of continuous variables between groups were performed using Student’s t-test or the Mann–Whitney U-test, as appropriate. Categorical variables were compared using the chi-squared test or Fisher’s exact test. Pearson and partial correlation analyses were conducted to assess associations among continuous variables with a normal distribution, and Spearman’s rank correlation was used for continuous variables without a normal distribution or for categorical variables. ROC curve analysis was conducted to evaluate the discriminative ability of serum TMAO levels in identifying patients with SLE with CVD. For microbiome analysis, raw sequencing reads were denoised and amplicon sequence variants were generated using DADA2 R package (v1.20.0), a high-resolution amplicon denoising algorithm. Subsequent analyses were performed using QIIME2 (Quantitative Insights Into Microbial Ecology 2, v2021.11). Taxonomic assignment was conducted using the Phyloseq R package (v1.50.0). Alpha-diversity indices were calculated using the diversity plugin in QIIME2, and group differences were assessed using the Wilcoxon rank-sum test. Beta-diversity was evaluated based on Bray–Curtis distances and tested for statistical significance using permutational multivariate analysis of variance. Differential abundance of microbial taxa between groups was analyzed using the DESeq2 R package (v1.46.0). Statistical analyses were conducted using the R software (Windows 4.4.2; The R Foundation for Statistical Computing, Vienna, Austria) or SPSS software (version 29.0; IBM Corporation, Armonk, NY, USA). Statistical significance was defined as a *P*-value < 0.05.

## Results

### Baseline characteristics of the study participants

The baseline clinical characteristics of the 157 patients with SLE and 50 HCs at the time of sample collection are presented in Table 1. There were no statistically significant differences between the two groups in terms of age, sex, or body mass index (BMI). The median disease duration among patients with SLE was 112 months (interquartile range [IQR] 45.75–221.5). The median serum levels of C3, C4, and anti-DNA antibodies were 81 mg/dL (IQR 68–93), 16.9 mg/dL (IQR 9.5–21.6), and 21.73 IU/mL (IQR 5.28–73.03), respectively. The median Systemic Lupus Erythematosus Disease Activity Index 2000 (SLEDAI-2 K) score was 4 (IQR 2–7), and 43 patients (27.4%) had a score of ≥ 7. The median Systemic Lupus International Collaborating Clinics/American College of Rheumatology (ACR) damage index (SLICC/ACR DI) was 1 (IQR 0–1). Lupus nephritis was identified in 64 patients (40.8%), and 67 patients (42.7%) had comorbid CVD. Steroids were administered to 134 patients (85.4%) at a median daily dose of 2.5 mg (IQR 2.5–5), and hydroxychloroquine was prescribed to 139 patients (88.5%).

**Table 1:** Baseline clinical characteristics of included patients with SLE and healthy controls.

Variables	Healthy controls (*n* = 50)	Patients with SLE (*n* = 157)	*P*-value
Age, years	40 [32–44]	40 [31–49]	0.233
Sex, female	45 (90)	141 (89.8)	1.000
Body mass index, kg/m^2^	22.7 [20.1–24.7]	21.8 [19.5–23.8]	0.858
eGFR, ml/min/1.73 m^2^		106 [88.8–116]	
Disease duration, months		112 [45.75–221.5]	
C3, mg/dL		81 [68–93]	
C4, mg/dL		16.9 [9.5–21.6]	
Anti-DNA antibody, IU/mL		21.73 [5.28–73.03]	
SLEDAI-2 K score		4 [2–7]	
≥7		43 (27.4)	
SLICC/ACR damage index		1 [0–1]	
Lupus nephritis		64 (40.8)	
Cardiovascular disease		67 (42.7)	
Steroid use		134 (85.4)	
Steroid dose, mg/day		2.5 [2.5–5]	
Hydroxychloroquine use		139 (88.5)	

*P < 0.05, **P < 0.01. Data are presented as medians [interquartile ranges] or n (%). SLE, systemic lupus erythematosus; eGFR, estimated glomerular filtration rate; C3, complement 3; C4, complement 4; SLEDAI-2 K, SLE Disease Activity Index 2000; SLICC/ACR, Systemic Lupus International Collaborating Clinics/American College of Rheumatology.

### Serum TMAO and clinical parameters

**Fig. 1:**
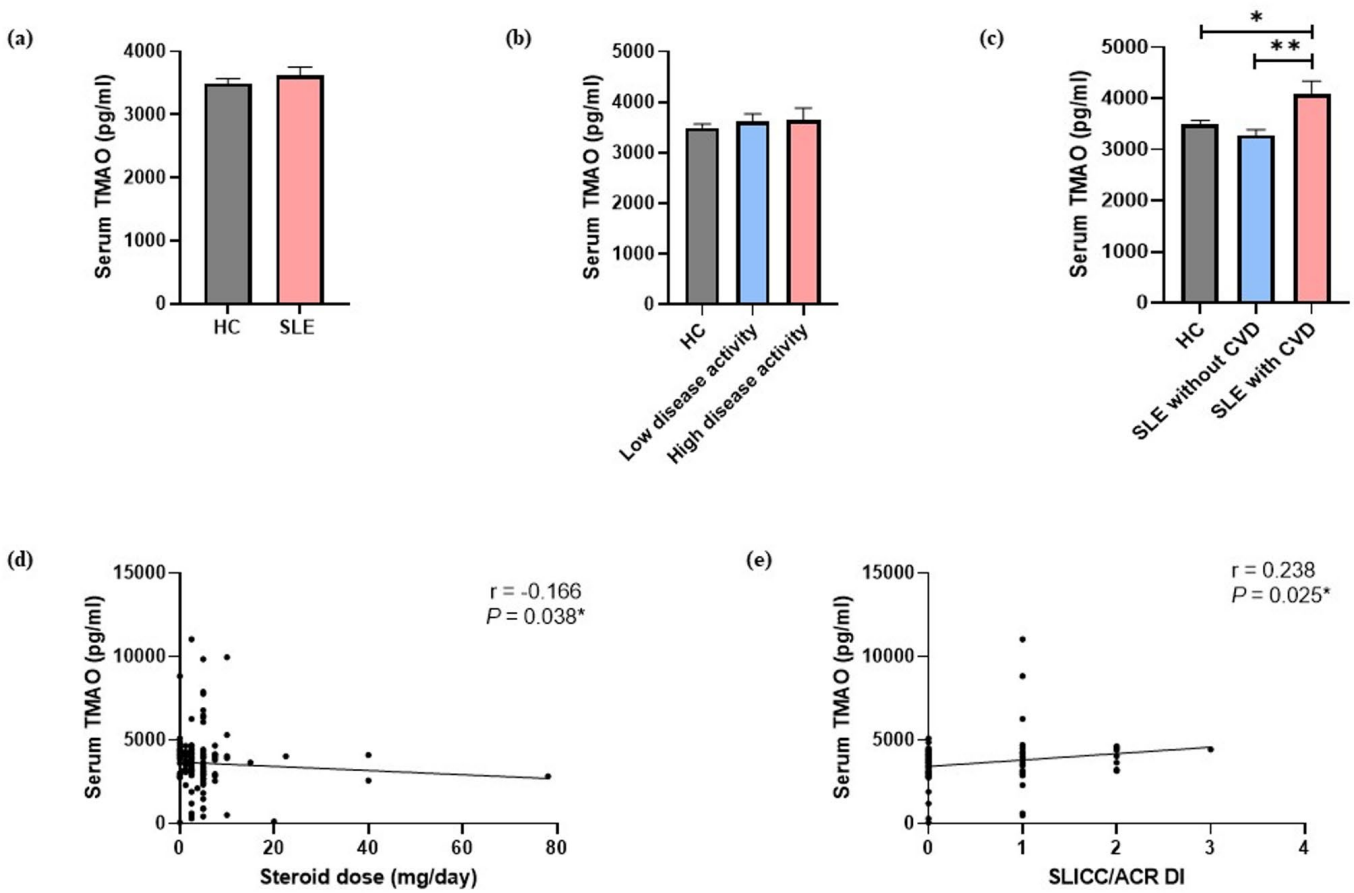
Serum TMAO levels in patients with SLE and healthy controls (HCs). (**a**) Comparison of serum TMAO levels between HCs and patients with SLE, (**b**) serum TMAO levels in patients with SLE stratified by disease activity, and (**c**) serum TMAO levels in patients with SLE with or without comorbid cardiovascular disease (CVD) were analyzed using Student’s t-test. (**d**,**e**) Spearman’s correlation between serum TMAO levels and (**d**) steroid dose, and (**e**) SLICC/ACR DI in patients receiving ≤ 2.5 mg/day of prednisolone. TMAO, trimethylamine N-oxide; SLE, systemic lupus erythematosus; HCs, healthy controls; CVD, cardiovascular disease; SLICC/ACR DI, Systemic Lupus International Collaborating Clinics/American College of Rheumatology damage index. *P < 0.05, **P < 0.01.

The serum TMAO levels in patients with SLE and HCs, along with their associations with relevant clinical parameters, are presented in Fig. 1. The serum TMAO levels did not show a statistically significant difference between patients with SLE and HCs (3621.97 ± 129.72 pg/mL vs. 3493.59 ± 76.85 pg/mL; *P* = 0.396; Fig. 1a). Stratification by disease activity showed a progressive increase in the serum TMAO levels from HCs to patients with SLE with low disease activity (SLEDAI-2 K < 7; 3612.79 ± 154.54 pg/mL) and high disease activity (SLEDAI-2 K ≥ 7; 3646.32 ± 240.52 pg/mL); however, the differences did not reach statistical significance (Fig. 1b). Subgroup analysis based on comorbid CVD revealed that patients with SLE with CVD (4085.86 ± 252.54 pg/mL) had significantly higher serum TMAO levels than HCs (*P* = 0.028) and patients with SLE without CVD (3276.63 ± 144.48 pg/mL; *P* = 0.004) (Fig. 1c). The receiver operating characteristic (ROC) curve discriminating between patients with SLE with CVD and patients with SLE without CVD or HCs revealed an area under the curve (AUC) of 0.6226 (95% CI 0.5313–0.7139, *P* = 0.004). The discriminative performance of the ROC curve improved slightly when the analysis was restricted to patients with SLE with and without CVD, yielding an AUC of 0.6343 (95% CI 0.5427–0.7260, *P* = 0.004) (Supplementary Fig. S1). A significant negative correlation was observed between serum TMAO levels and the concurrent steroid dose (*r* = − 0.166, *P* = 0.038) (Fig. 1d). As anticipated, steroid dose was significantly positively correlated with the SLICC/ACR DI (*r* = 0.325, *P* < 0.001). To minimize the potential confounding effect of steroid use, a subgroup analysis was conducted including only 89 patients with SLE receiving ≤ 2.5 mg/day of prednisolone. Within this subgroup, the serum TMAO levels showed a significant positive correlation with the SLICC/ACR DI (*r* = 0.238, *P* = 0.025) (Fig. 1e).

### TMAO-related fecal metabolites

**Fig. 2:**
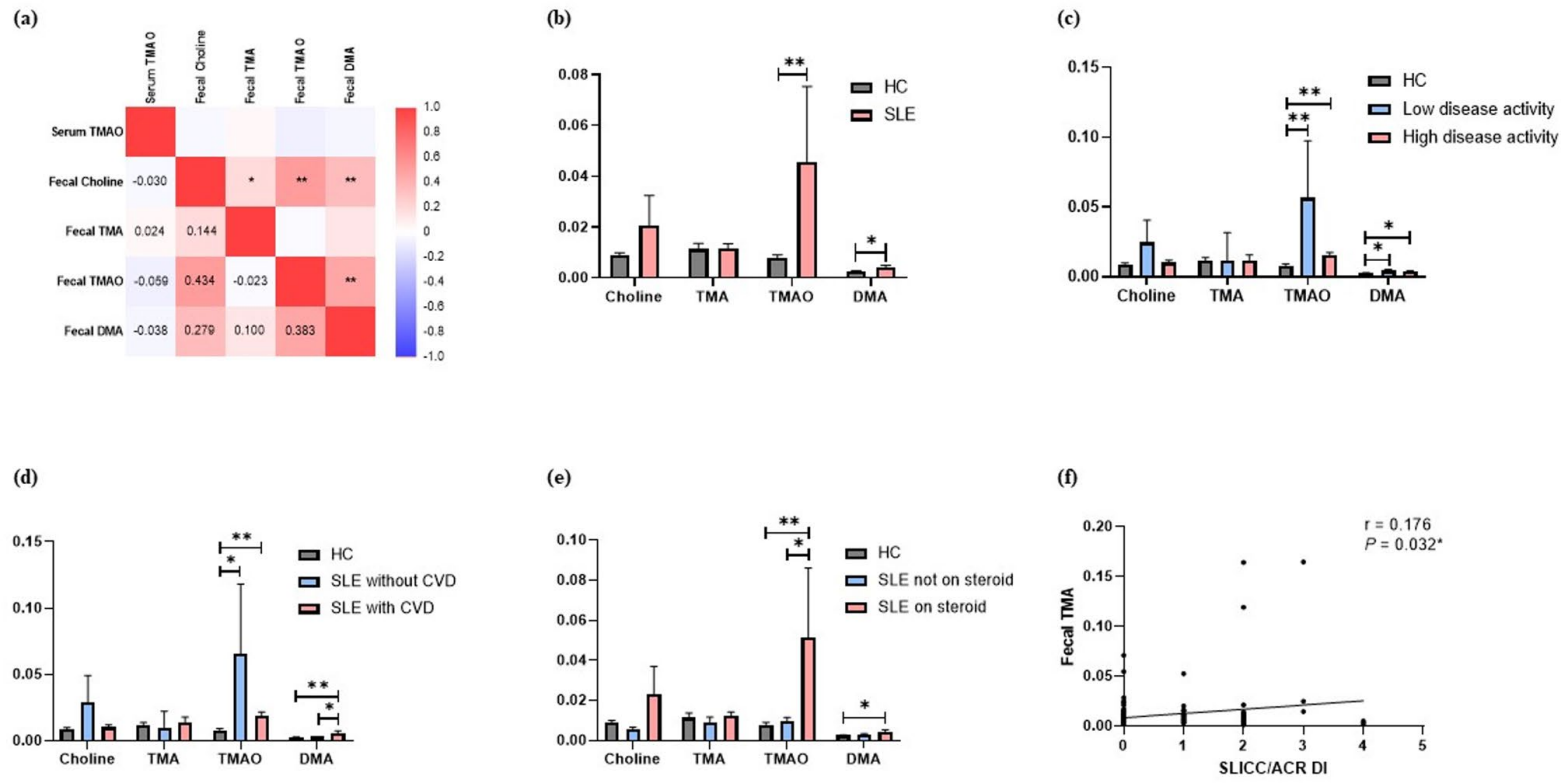
TMAO-related fecal metabolite levels in patients with SLE and healthy controls (HCs). (**a**) Spearman’s correlation heatmap showing correlation coefficients and *P*-values between serum TMAO and fecal metabolites. (**b**) Comparison of fecal metabolite levels between HCs and patients with SLE, and (**c**–**e**) fecal metabolite levels in patients with SLE stratified by (**c**) disease activity, (**d**) comorbid cardiovascular disease (CVD), and (**e**) steroid use were analyzed using the Mann–Whitney U-test. (**f**) Pearson partial correlation (adjusted for choline) between fecal TMA levels and the SLICC/ACR DI. TMA, trimethylamine; TMAO, trimethylamine N-oxide; DMA, dimethylamine; SLE, systemic lupus erythematosus; HC, healthy controls; CVD, cardiovascular disease; SLICC/ACR DI, Systemic Lupus International Collaborating Clinics/American College of Rheumatology damage index. *P < 0.05, **P < 0.01.

In the correlation analysis, fecal TMA (*r* = 0.144, *P* = 0.043), fecal TMAO (*r* = 0.434, *P* < 0.001), and fecal DMA (*r* = 0.279, *P* < 0.001) levels were all positively correlated with fecal choline, the initial precursor. Additionally, the fecal DMA levels were positively correlated with the fecal TMAO levels (*r* = 0.383, *P* < 0.001). There was no significant association between the serum TMAO and any fecal metabolites (Fig. 2a).

According to fecal metabolite comparison analyses, patients with SLE exhibited significantly higher fecal TMAO (0.045 ± 0.030 vs. 0.008 ± 0.001; *P* < 0.001) and DMA levels (0.004 ± 0.001 vs. 0.003 ± 0.000; *P* = 0.018) compared to HCs (Fig. 2b). Subgroup analyses based on SLE disease activity, presence of comorbid CVD, and steroid use are presented in Fig. 2c–e, respectively. The fecal TMA levels, adjusted for precursor choline, showed a significant positive correlation with the SLICC/ACR DI in patients with SLE (*r* = 0.176, *P* = 0.032; Fig. 2f).

**Fig. 3:**
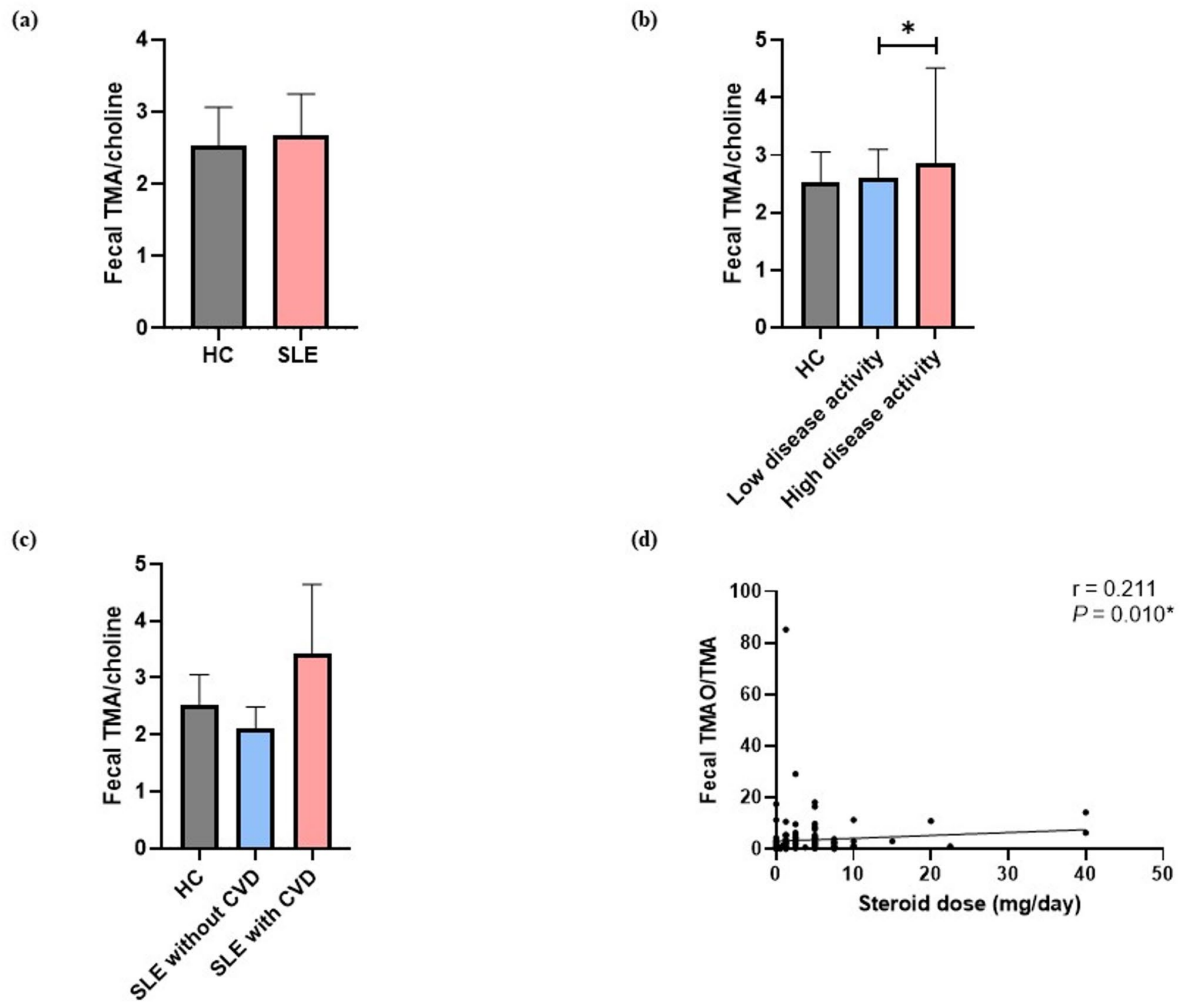
Fecal metabolite ratios reflecting enzyme activity in patients with SLE and healthy controls (HCs). (**a**) Comparison of fecal TMA/choline ratios between HCs and patients with SLE, and (**b**,**c**) fecal TMA/choline ratios in patients with SLE stratified by (**b**) disease activity and (**c**) comorbid cardiovascular disease (CVD) were analyzed using the Mann–Whitney U-test. (**d**) Pearson partial correlation (adjusted for choline) between fecal TMAO/TMA ratios and steroid dose. SLE, systemic lupus erythematosus; HCs, healthy controls; TMA, trimethylamine; TMAO, trimethylamine N-oxide; CVD, cardiovascular disease. *P < 0.05, **P < 0.01.

In the gut, TMA lyase metabolizes choline into TMA^28^; therefore, the fecal TMA/choline ratio likely reflects TMA lyase activity. Fecal TMA/choline ratios did not show a statistically significant difference between patients with SLE and HCs (2.676 ± 0.569 vs. 2.531 ± 0.526; *P* = 0.816; Fig. 3a). Subgroup analysis according to disease activity revealed that patients with SLE with high disease activity (SLEDAI-2 K ≥ 7; 2.862 ± 1.656) exhibited significantly higher fecal TMA/choline ratios compared to those with low disease activity (SLEDAI-2 K < 7; 2.608 ± 0.492; *P* = 0.039; Fig. 3b). Similarly, patients with SLE with comorbid CVD (3.419 ± 1.227) showed a trend toward higher fecal TMA/choline ratios compared to HCs and patients with SLE without CVD (2.116 ± 0.374), although the differences did not reach statistical significance (Fig. 3c). Notably, as shown in Fig. 2e, patients with SLE receiving steroid (0.051 ± 0.035) exhibited significantly higher fecal TMAO levels compared to HCs (0.008 ± 0.001; *P* < 0.001) and patients with SLE not receiving steroids (0.009 ± 0.002; *P* = 0.040). Fecal TMAO is produced in the gut via the oxidation of TMA by TMAO monooxygenase and can be reduced back to TMA by TMAO reductase. Accordingly, the fecal TMAO/TMA ratio likely reflects the enzymatic activity of TMAO monooxygenase or the reverse activity of TMAO reductase. After adjustment for the precursor choline, the fecal TMAO/TMA ratio showed a significant positive correlation with steroid dose (*r* = 0.211, *P* = 0.010; Fig. 3d).

### Gut microbiome in patients with SLE with and without comorbid CVD

**Fig. 4:**
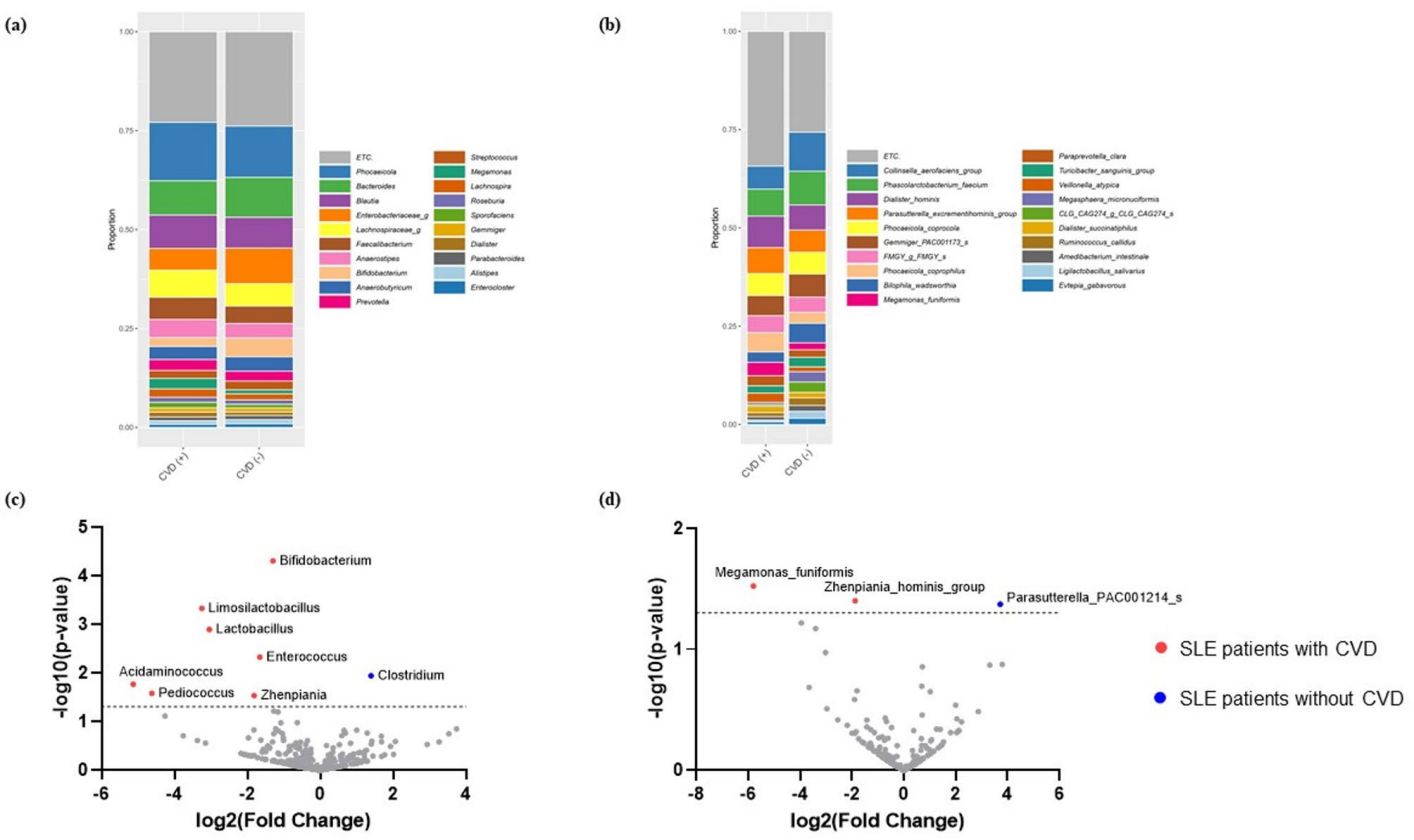
Gut microbiota composition in patients with SLE with and without comorbid cardiovascular disease (CVD). Stacked bar graphs showing the average relative abundance of the top 20 microbial (**a**) genera and (**b**) species, respectively. Lower-abundance taxa are grouped under the “ETC” category. Each bar represents the average microbial composition within each group, with distinct colors indicating different genera or species. (**c**,**d**) Volcano plots displaying DESeq2-based differential abundance analysis results at the (**c**) genus and (**d**) species levels between patients with SLE with and without CVD. Each point represents a bacterial genus or species, with the x-axis indicating log2 fold change (with CVD vs. without CVD) and the y-axis indicating − log10-transformed adjusted *P*-values. Taxa with an adjusted *P*-value ≤ 0.05 and an absolute log2 fold change > 1 were considered significantly differentially abundant. Taxa enriched in patients with SLE with CVD are shown in red; those enriched in patients without CVD are shown in blue; non-significant taxa are shown in gray. SLE, systemic lupus erythematosus; CVD, cardiovascular disease.

Gut microbiome analysis among patients with SLE with and without comorbid CVD revealed no significant differences in alpha or beta diversity between the two groups (Supplementary Fig. S2). Gut microbiota composition at the genus and species levels is presented in Fig. 4a,b, respectively. Differential abundance analysis using DESeq2 is presented in Fig. 4c,d as volcano plots. At the genus level, patients with SLE with comorbid CVD exhibited significantly increased abundances of *Bifidobacterium* (*P* < 0.001), *Limosilactobacillus* (*P* = 0.0005), *Lactobacillus* (*P* = 0.0013), *Enterococcus* (*P* = 0.0048), *Acidaminococcus* (*P* = 0.0172), *Pediococcus* (*P* = 0.0266), and *Zhenpiania* (*P* = 0.0293), whereas *Clostridium* (*P* = 0.0115) was significantly more abundant in patients with SLE without comorbid CVD (Fig. 4c). At the species level, *Megamonas funiformis* (*P* = 0.0300) and the *Zhenpiania hominis group* (*P* = 0.0398) were significantly enriched in patients with SLE with comorbid CVD, whereas *Parasutterella PAC001214 s* (*P* = 0.0424) was more abundant in those without comorbid CVD (Fig. 4d). DESeq2 analysis comparing HCs and patients with SLE at the genus and species levels is presented in Supplementary Fig. S3. Among the taxa significantly enriched in patients with SLE relative to HCs, genera that were further significantly enriched in patients with SLE with comorbid CVD compared to those without were *Limosilactobacillus*, *Lactobacillus*, and *Pediococcus*. Despite this enrichment, correlations between the abundance of these genera and serum TMAO, fecal TMA/choline ratios, and fecal TMAO/TMA ratios were not statistically significant (all *P* > 0.05, Supplementary Table S1). PICRUSt2-based functional prediction suggested enrichment of functions related to signal transduction, carbohydrate metabolism, cell wall-associated enzymatic activity, and membrane transport in patients with SLE with CVD, whereas nitrate reductase-related activity was relatively enriched in patients with SLE without CVD (Supplementary Fig. S4).

## Discussion

In this study, the serum TMAO levels were higher in patients with SLE with comorbid CVD compared to HCs and patients with SLE without CVD. The serum TMAO and fecal TMA levels were positively correlated with the SLICC/ACR DI. In contrast, the serum TMAO levels and the TMAO reductase activity index were negatively correlated with steroid dose. Additionally, distinct gut microbiota compositions were observed between patients with SLE with and without CVD. At the genus level, *Limosilactobacillus*, *Lactobacillus*, and *Pediococcus* were significantly enriched in patients with SLE compared to HCs and more abundant in patients with SLE with comorbid CVD than in those without.

Patients with SLE with CVD had significantly higher serum TMAO levels than HCs and patients with SLE without CVD, suggesting a potential association between elevated circulating TMAO levels and the presence of cardiovascular comorbidity in SLE. Previous studies have reported that TMAO contributes to CVD development through various mechanisms, including promotion of foam cell formation, impairment of reverse cholesterol transport, endothelial dysfunction via oxidative stress, platelet hyperreactivity, and vascular inflammation^29,30^. Although the present study does not demonstrate a causal relationship between circulating serum TMAO levels and the development of CVD, the observed association suggests that TMAO may be involved in cardiovascular pathogenesis in patients with SLE. Given the previously reported mechanisms^29,30^, elevated TMAO levels may contribute to CVD development in SLE through these pathways.

After controlling for confounding effects, the SLICC/ACR DI of patients with SLE showed a significant positive correlation with serum TMAO levels and its precursor, fecal TMA levels. While patients with SLE with high disease activity had significantly higher fecal TMA/choline ratios compared to those with low disease activity, no significant differences in serum TMAO or fecal TMA levels were observed between HCs and patients with SLE when stratified by disease activity. These findings suggest that serum TMAO and fecal TMA levels are more closely associated with accumulated organ damage than with active inflammation in SLE. Persistent inflammation can lead to tissue injury and organ damage^25^, with accumulated damage known to increase over time and predicts future mortality in patients with SLE^31^. As TMAO circulates systemically and contributes to persistent inflammation, elevated serum TMAO levels are likely to be more closely associated with the damage index, which reflects chronic inflammation, rather than with the disease activity index, which reflects transient active inflammation. Furthermore, as the SLICC/ACR DI includes a cardiovascular domain, the observed association between TMAO and the SLICC/ACR DI may partly reflect the previously reported association between TMAO and CVD.

Conversely, steroid dose showed a significant negative correlation with the serum TMAO levels among patients with SLE. Notably, patients with SLE receiving steroids exhibited significantly higher fecal TMAO levels compared to HCs and patients with SLE not receiving steroids. Additionally, the fecal TMAO/TMA ratio was positively correlated with steroid dose. These findings suggest that steroids may suppress serum TMAO production by reducing the fecal TMA levels, either through promoting the oxidation of fecal TMA to fecal TMAO or by inhibiting the reduction of fecal TMAO back to fecal TMA. The reduction in circulating TMAO levels may be attributable to the anti-inflammatory effects of steroids. Steroids have a bidirectional relationship with cardiovascular risk in patients with SLE^16^. Steroids suppress inflammation and modulate atherogenesis by reducing cytokine levels, and low-dose steroids have demonstrated plaque-stabilizing effects^32,33^. However, prolonged use of high-dose steroids has been associated with a dose-dependent increase in cardiovascular risk, paradoxically accelerating plaque formation^32,34^. In our study, the steroid dose in patients with SLE was low (median 2.5 mg/day), and the findings align with previous reports indicating that low-dose steroids exert anti-inflammatory effects and may reduce cardiovascular risk. TMAO may plausibly be involved as one of the mechanisms through which low-dose steroids contribute to cardiovascular risk reduction.

Differences in gut microbiome composition were observed between patients with SLE with and without comorbid CVD, in addition to differences between HCs and patients with SLE. Seventeen genera were significantly enriched in patients with SLE compared to HCs. Among these, *Limosilactobacillus*, *Lactobacillus*, and *Pediococcus* were further significantly increased in patients with SLE with comorbid CVD compared to those without. The lack of statistically significant correlations between the abundance of these genera and metabolite levels may be attributable to their relatively low abundance within the overall microbial community, which could limit the statistical power to detect direct associations. Although the role of *Lactobacillus* in patients with SLE as either a beneficial or harmful microbe remains inconclusive^35^, the plasma TMAO levels are reportedly positively correlated with *Lactobacillus* in an *Apoe*−/− mouse model^36^, and that urinary TMAO levels were significantly positively correlated with the abundance of *Lactobacillus* in children with early-stage chronic kidney disease^37^. For *Limosilactobacillus* and *Pediococcus*, existing reports are far more limited with respect to patients with SLE^38^ and their association with TMAO^39,40^. *Limosilactobacillus*, *Lactobacillus*, and *Pediococcus* all belong to the phylum *Firmicutes*, which has been largely reported to be decreased in patients with SLE compared to HCs^41–43^. However, *cutC* homologs, which are involved in the production of TMA from choline, are known to be more common in *Firmicutes* and relatively scarce in *Bacteroidetes*^44^. Several microbiome studies have reported an increased abundance of *Firmicutes* in patients with CVD^45–48^. Our study suggests that a phylum regarded as beneficial due to its overall reduction in patients relative to HCs may be considered harmful according to specific subgroups. Although further research is needed to elucidate the mechanisms through which the microbiome contributes to comorbid CVD in SLE, the present results shed light on the potential involvement of the gut microbiome in relation to TMAO.

This study has some limitations. First, it was conducted in a single-ethnicity Korean cohort, which may limit the generalizability of the findings. Future studies with larger, multiethnic cohorts are necessary. Second, TMAO levels were measured in a single serum and fecal sample from each participant, and serum TMAO levels were quantified using an ELISA-based assay. To minimize potential variability related to diet, serum and fecal samples were collected after a fasting period of at least 8 h. Although prior studies have reported that circulating TMAO levels are relatively stable over 12 months^49^, repeated measurements over time may provide a more accurate and comprehensive assessment. Third, the cross-sectional design precludes conclusions regarding causality or the directionality of associations between the gut microbiome, TMAO-related metabolites, and clinical parameters. Finally, dietary information was collected but not incorporated as covariates in the analysis because no dietary restrictions were imposed prior to fecal sampling and the information was primarily descriptive. Although fecal choline level-adjusted analyses were conducted to account for differences in precursor availability and minimize confounding, the lack of standardized dietary assessments remains a limitation that limits definitive causal interpretations regarding the observed metabolic and microbial differences. In addition, unmeasured factors, such as individual differences in gut permeability or FMO3 activity, may have influenced the results, as patients with SLE are known to exhibit increased intestinal permeability, also known as “leaky gut”^43^, and rare *FMO3* mutations reducing the activity of the enzyme have been reported^44^. Moreover, although PICRUSt2-based functional prediction in this study suggests that cardiovascular comorbidity in SLE may be associated with shifts in microbial functional potential, this approach cannot capture actual gene expression (e.g., cutC/D) or provide strain-level or pathway-specific resolution. Future studies using shotgun metagenomics, targeted qPCR, and direct functional analyses are needed to clarify the specific microbial mechanisms involved in TMAO metabolism.

Nonetheless, the present study is noteworthy as, to our knowledge, it is the first to provide a comprehensive analysis of TMAO, its precursor metabolites, and the gut microbiome in patients with SLE. Emerging evidence suggests that TMAO may play a contributory role in SLE pathogenesis. Untargeted metabolomic studies have reported elevated circulating TMAO levels in patients with SLE^18^, and experimental mouse models have demonstrated that TMAO promotes autoimmunity, oxidative stress, and endothelial dysfunction, whereas inhibition of TMA formation ameliorates these features^17^. As TMAO is a gut microbiota-derived metabolite, its role in SLE aligns with the well-established gut dysbiosis in SLE. Our findings further underscore a potential role for TMAO in SLE, particularly in relation to CVD, and indicate its possible relevance as a therapeutic target.

In conclusion, our study showed that TMAO appears to be associated with CVD and organ damage in patients with SLE. Steroids, as anti-inflammatory agents, are likely to be associated with reduced TMAO levels. Furthermore, the gut microbiome may play a contributory role in the underlying mechanisms.

## Supplementary Information

Below is the link to the electronic supplementary material.

Supplementary Material 1

Supplementary Material 2

Supplementary Material 3

Supplementary Material 4

Supplementary Material 5

## Data Availability

The dataset generated and/or analysed during the current study have been deposited in the Korean BioData Station (K-BDS) repository under accession number KAP241782. Due to funding conditions, the data are currently under restricted access but will become publicly accessible on 31 December 2027. Requests for access prior to the date may be addressed to the corresponding author.
