# Supplementary figures and images for: Clinical significance of gut microbiota-derived metabolite trimethylamine N-oxide in patients with systemic lupus erythematosus
Source: Sci Rep. 2026 May 22;16:23438. doi: 10.1038/s41598-026-53011-7 (PMC13408999; doi:10.1038/s41598-026-53011-7)

## Slide 1
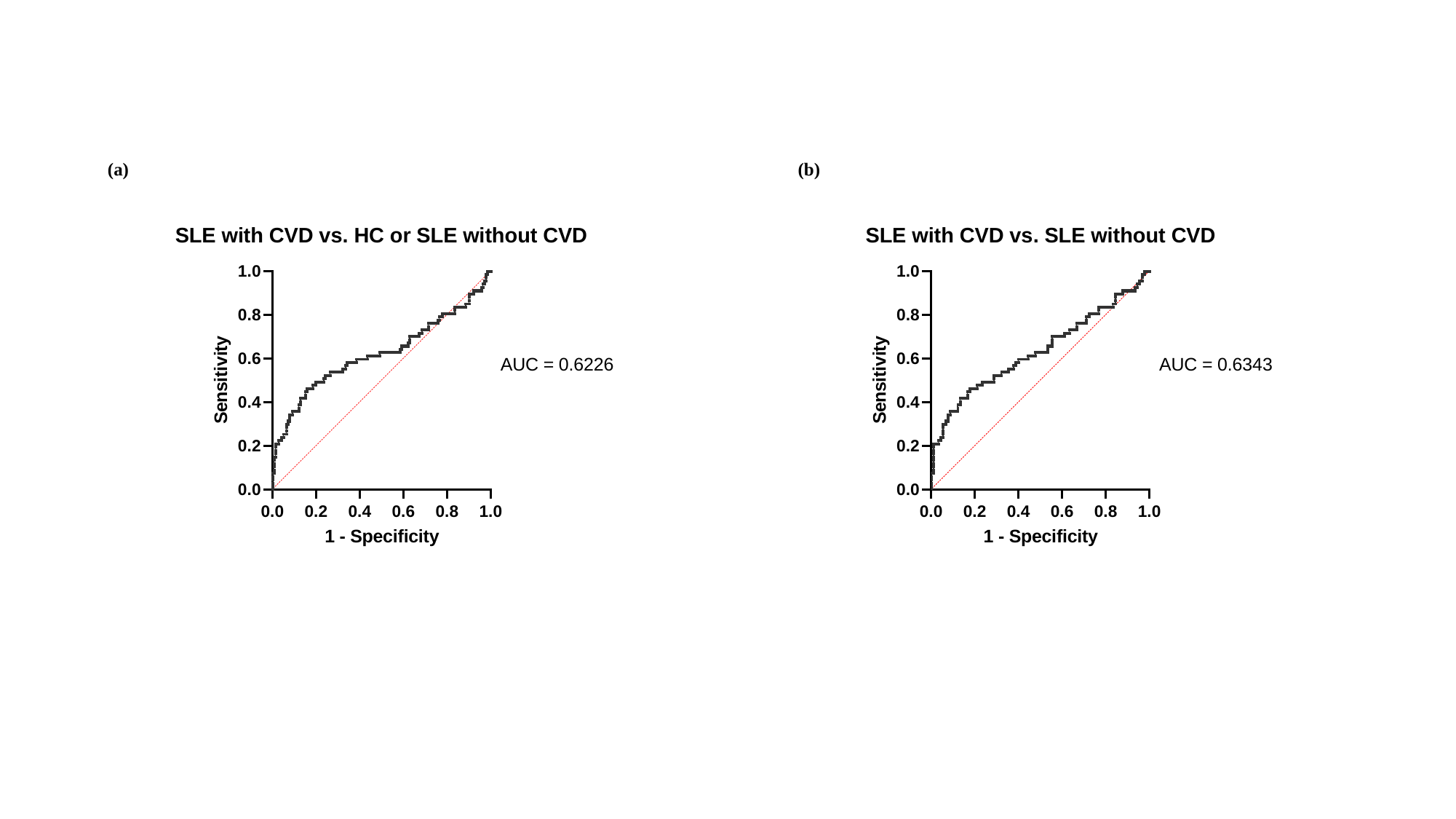

(a)
(b)

Supplement: Supplementary file 1 — Supplementary Material 1 [file 41598_2026_53011_MOESM1_ESM.pptx]

## Slide 1
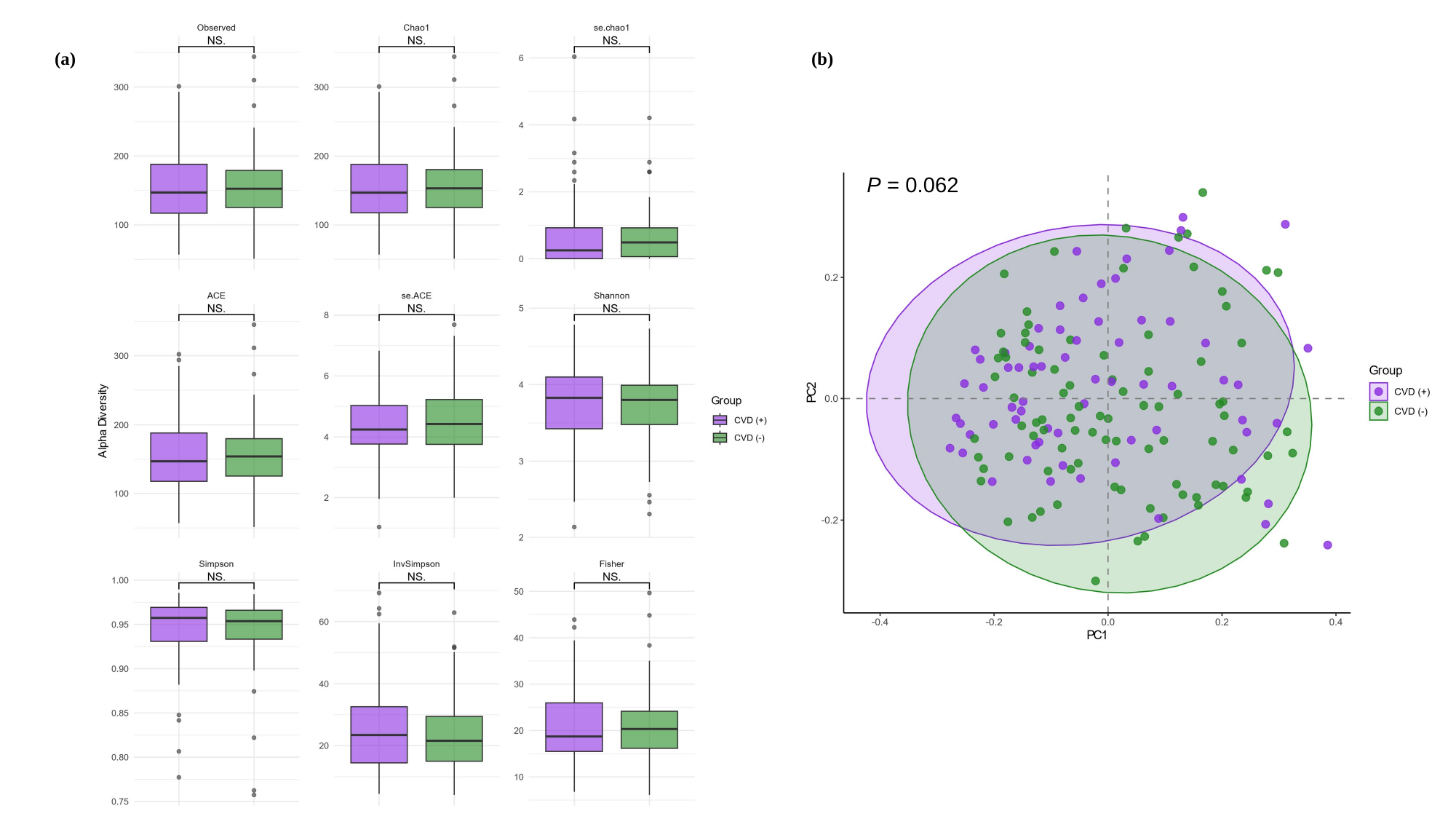

(b)
(a)
P = 0.062

Supplement: Supplementary file 2 — Supplementary Material 2 [file 41598_2026_53011_MOESM2_ESM.pptx]

## Slide 1
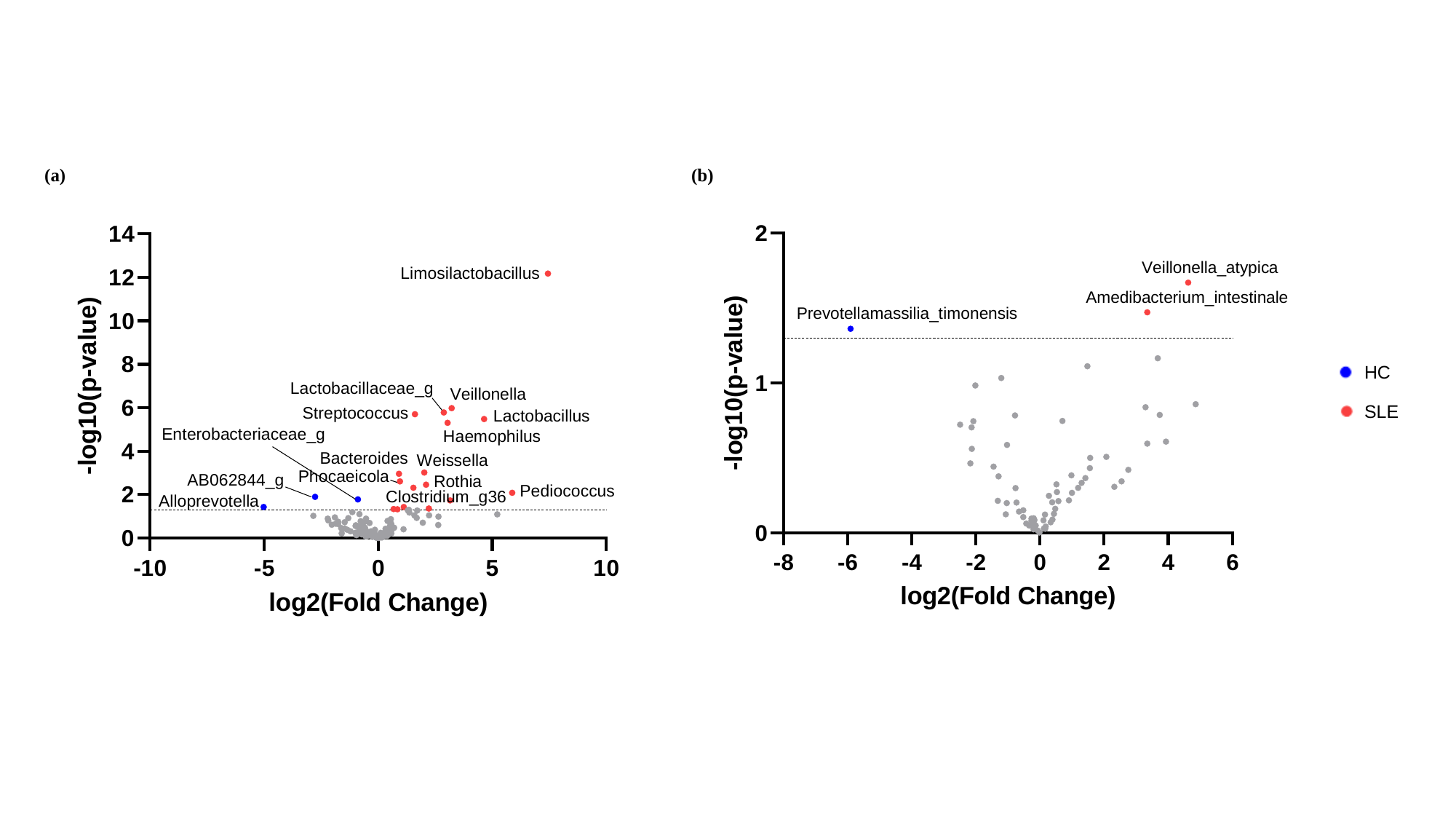

(b)
(a)
HC
SLE

Supplement: Supplementary file 3 — Supplementary Material 3 [file 41598_2026_53011_MOESM3_ESM.pptx]

## Slide 1
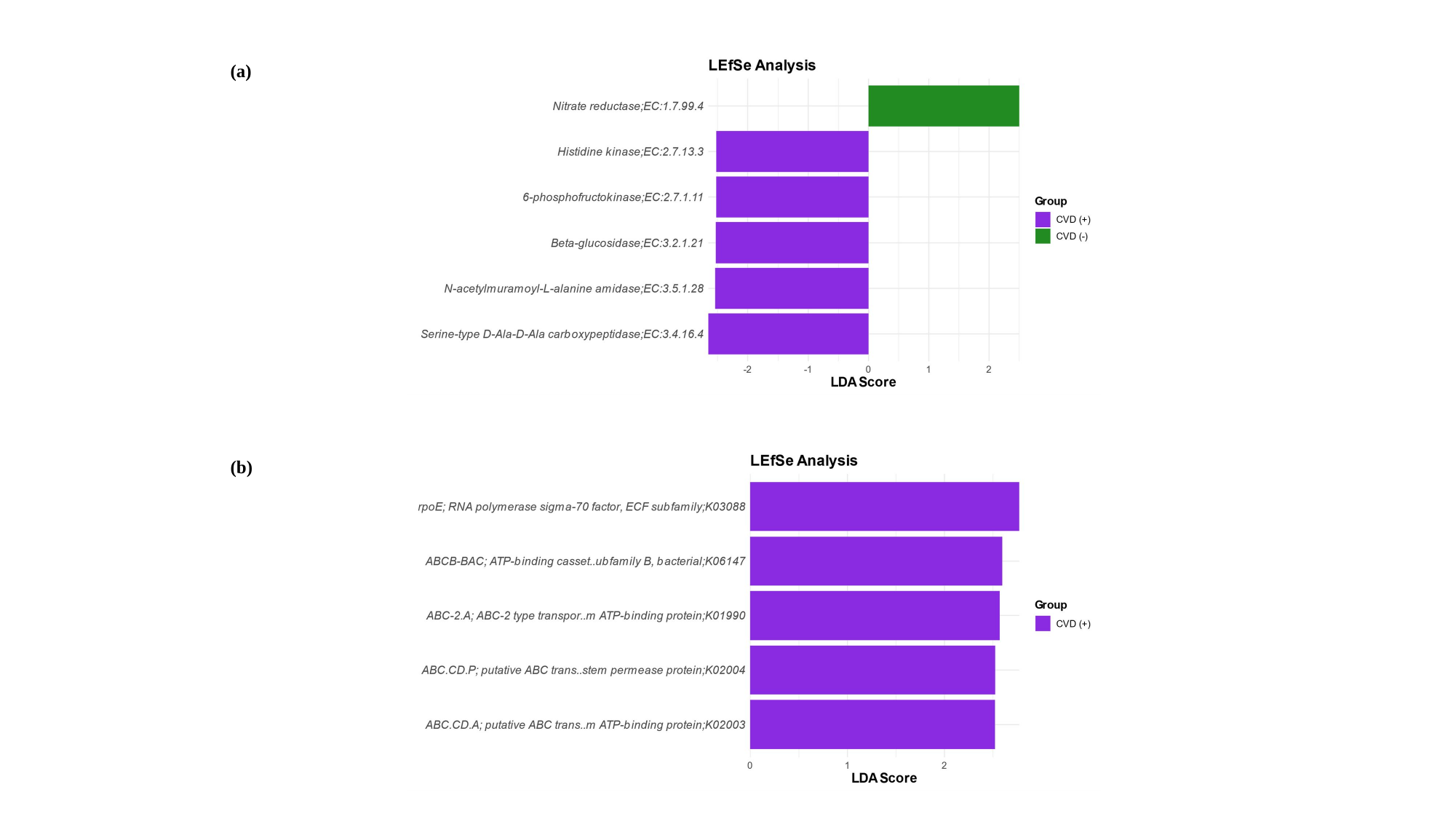

(a)
(b)

Supplement: Supplementary file 5 — Supplementary Material 5 [file 41598_2026_53011_MOESM5_ESM.pptx]
