# Supplementary material for: Clinical significance of gut microbiota-derived metabolite trimethylamine N-oxide in patients with systemic lupus erythematosus
Source: Sci Rep. 2026 May 22;16:23438. doi: 10.1038/s41598-026-53011-7 (PMC13408999; doi:10.1038/s41598-026-53011-7)
Supplement: Supplementary file 4 — Supplementary Material 4 [file 41598_2026_53011_MOESM4_ESM.docx]

**Supplementary Table S1.** Correlations between gut microbiota and TMAO-related metabolites in patients with SLE

| Taxa (Genus) | Metabolite | Spearman’s correlation coefficient (*r*) | *P*-value |
| --- | --- | --- | --- |
| *Lactobacillus* | Serum TMAO | -0.053 | 0.517 |
|  | Fecal TMA/choline ratio | 0.074 | 0.369 |
|  | Fecal TMAO/TMA ratio | -0.123 | 0.134 |
| *Limosilactobacillus* | Serum TMAO | -0.159 | 0.052 |
|  | Fecal TMA/choline ratio | 0.025 | 0.758 |
|  | Fecal TMAO/TMA ratio | -0.019 | 0.822 |
| *Pediococcus* | Serum TMAO | -0.047 | 0.572 |
|  | Fecal TMA/choline ratio | -0.151 | 0.066 |
|  | Fecal TMAO/TMA ratio | 0.147 | 0.074 |

^*^ *P* < 0.05, ^**^ *P* < 0.01. TMAO, Trimethylamine N-oxide; SLE, systemic lupus erythematosus; TMA, trimethylamine.
